# Linking body mass index and intelligence among Indian medical students

**DOI:** 10.6026/97320630018816

**Published:** 2022-09-30

**Authors:** Siktha Samanta, Yuvaraj Maria Francis, K Balaji, Gunapriya Raghunath, Samuel Sundar Doss, Madhan Krishnan, Shyamaladevi Babu

**Affiliations:** 1 Department of Biochemistry, Saveetha Medical College, Saveetha institute of medical and technical sciences, Thandalam, Chennai - 602 105, India; 2 Department of Anatomy, Saveetha Medical College, Saveetha institute of medical and technical sciences, Thandalam, Chennai- 602 105, India; 3 Department of Physiology, Saveetha Medical College, Saveetha institute of medical and technical sciences, Thandalam, Chennai - 602 105, India; 4 Faculty of Allied Health Sciences, Chettinad Hospital and Research Institute, Chettinad Academy of Research and Education, chengalpattu - 603103 India

**Keywords:** Adolescent, Body Mass Index, Correlation, Intelligence Quotient

## Abstract

The intelligence quotient (IQ) is a dependable measurement for intellectual functioning that reflects computable cognitive abilities. According to earlier cross-sectional studies designs, developing higher BMI related to decreased IQ in adolescents population. Therefore, it is of interest to determine the correlation between IQ and BMI. The Wechsler Intelligence Scale-IV was used to assess intelligence. Body Mass Index (weight in kg/height in metre squares) was calculated using height and weight. A questionnaire was developed after the elaborated discussion and circulated among the students. The data was then analyzed using Microsoft Excel 2019. The correlation among intelligent quotient and BMI was found to be positive: r = 0.447, N = 300, p<0.05. Data shows that the IQ score is moderately associated with BMI. However, the other factors such as, parental IQ, nutrition, and socioeconomic status is taken into account, this effect appears to differ.

## Background:

Globally, the younger population (aged 15 - 24 years) numbered 1.2 billion individuals or approximately one out of every six people. It is projected to exceed now almost 1.4 billion people by the year 2065, with a 13 percent rise [1]. The majority of young people are currently concentrated in southern Asia, including India. Physical and psychological well-being, parental involvement, learning environment, and financial resources are factors that determine the health of adolescent [2]. Development is a continuous process in which different factors (physical, motor, and environmental) are strongly associated and interrelated in a variety of ways. The quality of life is determined by the complex interplay of the genes, social and physical environments. Physical development is the geometric cell growth that is seen explicitly, whereas, cognitive development is more hard to ascertain than body development [3]. A fascinating study on a representative cohort on younger with enhanced intelligence quotient (IQ) youngsters revealed that they were increasing significance in motor performance than the regular populace [4]. There is substantial evidence that there is a relationship between poor intellectual capacity and being underweight. In rural India, the prevalence of low body mass indexed (BMI) children is four times greater (24%) than that of normal or high BMI children. This shows an intriguing interaction between environmental factors with motor and cognitive skills [5]. However a significant relationship between cognitive and motor functioning was not found. Another finding suggest that young people from higher-income families possess improved intellectual capabilities but relatively poor motor development, whereas young people from lower-income families possess improved motor function but relatively poor cognitive skills [6]. The concept of body and brain development, as well as research evidences, indicate that aspects of poverty, parental involvement, and nutritional sources influence young individuals BMI and IQ. So the aim of this study is to determine the BMI and IQ of the medical students and correlate it.

## Methods and Materials:

### Ethical Approval:

This cross-sectional study was done from June 2021 to October 2021 academic year after getting institutional ethical committee approval (Approval No: SMC/IEC/2021/03/144).

### Study Design and Data Set:

The participants were 300 medical students from phase I and II MBBS. The Wechsler Intelligence Scale-IV was used in this study to determine the students' Intelligence Quotient (IQ) scores, which is a highly reliable measure of general intellectual and cognitive functions. The average score on this scale was 100, with a standard deviation of 15 [7]. Weight and height were measured by automatic digital Weight Machine and fixed tape metre with a precision of 0.5 cm respectively. The formula used to find the BMI was kg/m2. All the measurements were done in the college campus, within the department itself. In accordance with the National Institutes of Health standards, the participants were categorized based on their BMI into underweight for BMI values of <18.5 kg/m2, normal weight for BMI values of 18.5∼23.0 kg/m2, overweight for BMI values of 23.0∼ 25.0 kg/m2, obese I for BMI values of 25.0∼30.0 kg/m2, and obese II for BMI values of ≥30.0 kg/m2. The questionnaire was designed with informed consent and 25 questions which enquire about the demographic profile, and socio-economic status.

### Statistical Analysis:

One-way analysis of variance (ANOVA) had been used in Microsoft Excel 2019 for statistics analysis. Furthermore, a graphical representation was done to investigate the relationship between BMI and Intelligent quotient as continuous variables, with p-values of 0.05 considered statistically significant.

## Results:

This study included 300 Phase I MBBS students, 169 (56.3) and 131 (43.6) percent were boys and girls respectively. The mean age, body mass index of these students were 17.50 ± 0.53, and 23.27 ± 3.25 respectively. The average intelligence quotient of the participants is 113.56 ± 8.14. The descriptive statistical analyses of these parameters are given in Table 1(see PDF).

Data shows that there no significant relation among Intelligence quotient and dietary habits, lifestyle factors, family socioeconomic background. Similarly, parental IQ and their education were inversely proportional to IQ and directly proportional to academic performance. Only twenty three percent of parental education level is significantly related to Intelligence quotient of the participants. Hence, the negative correlation between participant BMI and socioeconomic background, as well as Parental IQ, were not significant, and participant BMI did not correlate with academic performance significantly. The correlation among intelligent quotient and BMI was found to be positive: r = 0.447, N = 300, p<0.05. This correlation seems to be moderate and statistically significant. Because the intellectual capacity of men and women were determined by factors in a similar way, both sexes IQ were calculated together (Figure 1 - see PDF).

## Discussion:

In the past century, there was a regular and constant increase in fluid intelligence such as comprehension, reasoning and problem solving, and crystallized intelligence such as recalling stored knowledge and past experiences measured in several regions of the world which is known as the Flynn effect [8]. Study in Des Moines, Iowa, and Dumfries, Scotland compared participants aged over 100 years, for the Raven's Progressive Matrices test [9]. Intelligence quotient is found to be directly proportional to attendance among school children and inversely proportional to occurrence of illness. Educational qualification of the parents, socioeconomic status and academic performance are positively correlated with the children's intelligence quotient value [10-11].

Earlier research has yielded mixed findings. An Iranian study found a negative correlation between IQ and BMI among pre-school children [13]. In children under the age of five, there is no definite link between BMI and IQ in children aged below 5 years [14]. The review done in 2014, found that obesity in the younger age is associated with deficient intelligent quotient in childhood [15]. However, higher body mass index (BMI > 25) had negative correlation with cognition performance among the elderly population [16]. Few studies in the Netherlands found that intelligence was not associated to BMI among the school going children aged between 6 to 16 years old [17, 18]. Even though this study result gives important data on the relationship between BMI and IQ, it is worth noting that IQ is a non - modifiable risk factor that is hardly ever measured in the overall population. There are contradicting opinions about the correlation, and variation from region to region is to be anticipated. The processes that regulate BMI are complex and are affected by a combination of factors, including biological, socio cultural, environmental, hereditary, and lifestyle factors. None of these are fully understood. An observational study showed correlation between height and IQ can be merely due to result of a casual person to person difference. For example, the height of the individual varies up to adolescent age and cognitive performance is almost static throughout the lifespan [19, 20]. And there is considerable evidence for a deleterious relationship between weight and IQ, several other studies shows that overweight has conflicting effects on cognitive ability among adolescents [21, 22]. As a result, developing health consciousness program based on BMI and how it is correlated to intelligence is recommended. IQ can be assessed on a routine basis to educate individuals about the nutrition and balancing diet to live a healthy life. Moreover, individuals with limited cognitive function can be regularly evaluated for BMI to have a healthy society. The strength of this study are adolescent population was using for the first time to the best of our knowledge and test results that ensure data accuracy. The limitations are inability to account for potential confounders such as nutrition, cardio-respiratory fitness, and paternal level of intelligence.

## Conclusions:

This study shows that the majority of the participants had normal BMI. The BMI correlated with IQ, yet it did not show any influence on academic performance when the BMI alone was compared with the academic performance of the participants for a short duration, irrespective of the gender. In conclusion, body mass index have proven to be related to intelligent quotient in chronologically way. Even though this study found only moderately significant connection between BMI and intelligence, it is understood that, despite a significant sex difference adolescent period sue to hormonal changes, the BMI was unaffected by these changes. Since, this relationship between BMI and cognitive ability is challenging, more studies are required in this regard.
